# Contact Frequency, Travel Time, and Travel Costs for Patients with Rheumatoid Arthritis

**DOI:** 10.1155/2014/285951

**Published:** 2014-02-17

**Authors:** Jan Sørensen, Louise Linde, Merete Lund Hetland

**Affiliations:** ^1^Centre for Applied Health Services Research, University of Southern Denmark, J.B. Winsløws Vej 9B, 5000 Odense, Denmark; ^2^Center for Rheumatology and Spine Diseases, Glostrup Hospital, Denmark; ^3^Department of Clinical Medicine, Faculty of Health and Medical Sciences, University of Copenhagen, Denmark

## Abstract

*Objectives*. To investigate travel time, and travel cost related to contacts with health care providers for patients with rheumatoid arthritis (RA) during a three-month period. *Methods*. Patient-reported travel time and travel cost were obtained from 2847 patients with RA. Eleven outpatient clinics across Denmark recruited patients to the study. Data collected included frequency, travel time and travel costs for contacts at rheumatology outpatient clinics, other outpatient clinics, general practitioners, privately practicing medical specialists, inpatient hospitals and accident and emergency departments. *Results*. Over a 3-month period, patients with RA had on average 4.4 (sd 5.7) contacts with health care providers, of which 2.8 (sd 4.0) contacts were with rheumatology outpatient clinics. Private car and public travel were the most frequent modes of travel. The average patient spent 63 minutes and 13 € on travelling per contact, corresponding to a total of 4.6 hours and 56 € during the 3-month period. There was great variation in patient travel time and costs, but no statistically significant associations were found with clinical and sociodemographic characteristics. *Conclusion*. The results show that patients with RA spend private time and costs on travelling when they seek treatment. These findings are particularly important when analyzing social costs associated with RA.

## 1. Introduction

Patients' time and costs during illness and health care treatment are relevant aspects to include in a complete analysis of the social costs of health interventions [1]. Time and costs related to patients' travels from home to their health care providers are also relevant when the social implications of treatment options are assessed in, for example, cost-effectiveness analyses. Travel time and costs vary for the individual patient depending on the type of treatment provided, the frequency of contacts with health care providers, the traveled distance, and the mode of travel. While the need to include travel time and costs is widely accepted, details about how travel time and costs should be included have not been resolved [2–4].

A number of studies have investigated the travel time and costs for patients who are invited to attend hospital clinics as part of screening programs for, for example, cervical cancer [5], colorectal cancer [6], breast cancer [7], aortic aneurysm [8], and diabetic retinopathy [9], and for services that require frequent hospital attendance, for example, anticoagulation management [10–12].

Rheumatic arthritis (RA) is a chronic disease that requires life-long treatment and frequent contacts with health care providers to monitor and adjust medical treatment [13]. Treatment options that require frequent hospital visits may impose nontrivial travel time and costs on patients. However, no data on travel time and cost for RA patients have been reported in the scientific literature, although patients' time and travel costs have been considered in a few economic evaluations of RA interventions, for example [14]. An often-used strategy is to include patients' travel time and costs as a simple average without any attempt to obtain detailed information of the variation among individual patients' travel time and costs.

The objective of this study was to explore the frequency of contacts with different types of health care providers for Danish outpatients in care for RA, and assess average travel time and costs during a three-month period. As part of this study, we developed a strategy to obtain information from patients on their travel time and costs. A secondary study objective was to assess the strengths and weaknesses of this strategy.

## 2. Material and Methods

### 2.1. Patients and Data Collection

Patients with rheumatoid arthritis as defined by the ACR 1987 criteria were identified among consecutive patients attending 11 Danish rheumatology outpatient clinics between July 2006 and July 2007. Relevant patients were invited to participate in a patient-reported questionnaire study aimed at describing health-related quality of life, resource use, and time and costs spent on seeking health care treatment [15]. The participating departments consisted of a mix of university and local hospitals distributed across the country. Clinical staff at the participating clinics identified consecutive patients and administered a questionnaire booklet that patients were encouraged to complete and return during the visit at the outpatient clinic. Patients who agreed to complete the booklet were given a prepaid envelope to return it by mail or were allowed to return the questionnaire to the clinical staff. Each participating patient completed the questionnaire once. Using the personal identification number, we were able to ensure that only the data from the first returned questionnaire were included in the analysis.

In addition to information about travel time and costs, the questionnaire booklet asked for data on various social and life style factors including self-assessment of current health status using a global health score on a visual analogue scale (VAS), the health assessment questionnaire (HAQ) [16], and the EQ-5D [17]. In addition, clinical staff recorded information about the patient's clinical status including disease duration, disease activity (DAS-28 score and serum C-reactive protein (CRP) level), and use of RA medication.

### 2.2. Travel Time and Costs Questionnaire

**Figure 1:**
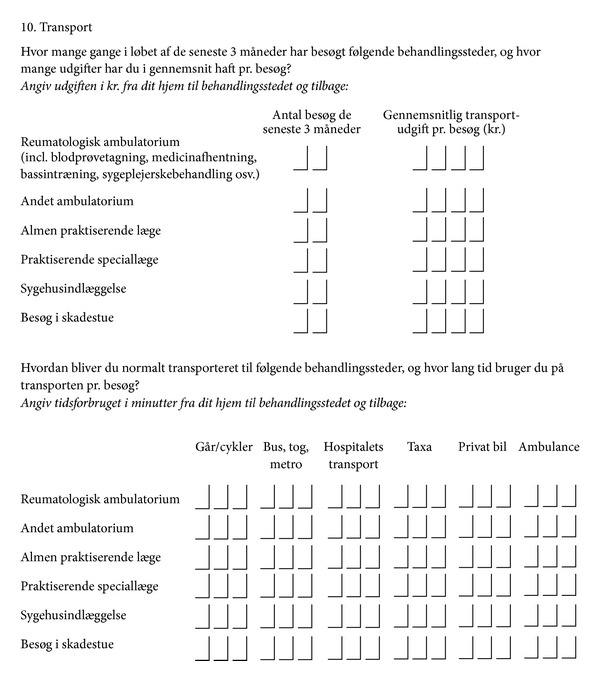
Travel Questionnaire (in Danish).

This part of the questionnaire consisted of one page with two sections of questions (see Figure 1). The Danish phrasing of questions was inspired by recommendations by a UK working party on patient-reported costs (Figure 1) [3]. The first section asked the question: “How many times during the last 3 months have you visited the following health care providers, and what did it cost you on average per visit? Please provide the cost in DKK from your home to the place of treatment and return.”

The following six types of health care providers were specified (one row for each):

- rheumatology outpatient clinic (for blood tests, collection of medication, training and contacts with nurses, etc.),
- other outpatient clinics,
- general practitioner,
- privately practicing medical specialist,
- hospital admission,
- emergency department.

For each provider, there were two columns with the following headings: Number of visits during the last three months; Average transport cost per visit (DKK).

The second section asked: “How do you normally travel to the following health care providers and how long time do you usually spend on travel per visit? Please state the time used in minutes from your home to the treatment site and return.”

The same six types of health care providers were specified and columns were provided to respond specifically to each of six different modes of travel (walking/biking; bus, train, metro (i.e., public transport); hospital-provided transport; taxi; private car; ambulance).

The questionnaire was pilot-tested in a small, convenient sample and slightly edited for clarity before the main study.

### 2.3. Data Coding

Personal and clinical data were categorized according to our previous analyses [15]. Briefly, age was categorized in three groups as 50 years or younger, 51–75 years, or 76 years or older. Cohabiting was categorized as living alone (single) or cohabiting; residential area was categorized as rural villages/smaller cities or larger cities; education was categorized according to duration as none/short (10 years or less), medium (11–13 years), or long (13+ years); body mass index (BMI) as underweight (<18.5), normal (18.5–25), overweight (25–35), or obese (>35). Labor market attachment was answered according to 10 categories and then recoded as in or out of the labor market. Disease duration was categorized as 0–2 years, 3–10 years, or 10+ years according to the date of first diagnosis and date of questionnaire completion; CRP levels were categorized as normal or elevated; biological treatment was classified as Yes or No. The scores of patient Global VAS and the EQ-5D VAS ranged from 0 to 100. A low score on Global VAS and a high EQ-5D VAS score indicated good health. The EQ-5D index was scored according to the standard Danish procedure [18]. Scores for individuals with more than one missing EQ-5D item were set to missing.

All cost data were uplifted from 2006-2007 price level to 2013 price level by multiplying the reported cost data by the ratio of the national consumer price index published by Statistics Denmark (http://www.statistikbanken.dk/pris6) January 2007 (112.4) and June 2013 (130.0). The national currency was converted to Euro assuming a currency rate of 100 DKK ~ 13.33 € (1 *€* ~ 7.50 DKK).

Some patients presumably misunderstood whether they should include the latest visit at the outpatient clinic in the three-month number of visits at a rheumatology outpatient clinic and provided a zero or missing number; thus, 35 respondents replied 0 and for 44 respondents a value was missing, but the patient had provided valid information about average travel costs. A total of 392 respondents did not provide valid travel expenses but provided valid information about mode of travel to the rheumatology outpatient clinic. Further 192 respondents did not provide any information about the number of visits, travel expenses, or mode of travel to the rheumatology outpatient clinic but provided other data in relation to travel. Since a visit to the rheumatology outpatient clinic was a condition for participating in the study, we replaced all missing numbers of rheumatology outpatient visits with a conservative one (“1”) visit.

Missing information about visits to other outpatient clinics, general practice, privately practicing specialists, and hospital admissions and visits at an emergency department was interpreted as zero visits.

Some patients who provided a valid number of visits did not report their average travel expenses. In the reporting of the raw data we present only the provided information (i.e., only responses with nonmissing items). In the analysis of travel costs, missing information about travel costs was replaced by the mean costs calculated from those who had reported a valid number of visits and a valid cost.

All numbers of visits were within the expected range (max 90 visits, that is, once a day over 3 months). We considered replacing or deleting the single response indicating 90 visits and the three responses indicating 60 visits but decided against this and retained the actual responses.

Travel expenses over 100 € (*n* = 23) were inspected more closely. Some of these related to travel in private cars over long distances. However, the seven patients who provided the highest travel expenses (up till 500 €) provided no information about travel time. These data appeared unrealistic and most likely due to the patient misunderstanding the question. As these few “extreme” observations had undesirable influences on the estimated mean figures, we replaced the seven observations with travel expenses over 200 € with the population mean.

### 2.4. Statistical Analysis

The average number of visits at different health care providers and the average travel time and costs per return visit are reported as number of valid responses, mean, standard deviation, and 25, 50, and 75 percentiles.

Modes of travel and time are reported as the number and proportion of individuals who use each of the different travel modes and the average travel time for those who use the particular travel mode. We aggregated the reported travel times for each individual and report the total travel time for those who have reported any travel data to the destination.

We examined differences in travel time and costs by gender, age, cohabiting status, residential area, education, labor market attachment, disease duration, CRP level, BMI, and biological treatment by bivariate comparisons using parametric tests. As neither the travel time nor the costs were normally distributed due to many individuals with low costs/time and few with very large costs/time, we reanalyzed the data using nonparametric tests, but these produced similar results. Given the high number of observations and the preference for reporting cost data as averages, we report here only the applied parametric tests (*t*-test/anova).

### 2.5. Ethics

Scientific ethical approval was not required for this survey study according to Danish regulations. The data collection was registered with the Danish Data Protection Agency.

## 3. Results

Of the 3704 patients invited to complete the questionnaire booklet, 549 patients (15%) did not return the booklet and 308 (8%) patients did not complete the travel questions. The analysis was thus based on replies from 2847 patients (77% of those invited and 90% of those who returned the questionnaire).

The characteristics of the study sample are compared with the nonresponders in Table 1. Chi-squared tests indicated that the study sample was younger than both groups of nonresponders (*P* < 0.01), more were married/cohabiting (*P* < 0.01), living within a city area (*P* = 0.02), had longer education (*P* < 0.01), and were in the labor market (*P* < 0.01). More individuals in the study sample had normal CRP levels (*P* = 0.04) and received treatment with biological medication (*P* = 0.01). *t*-tests indicated that the study population reported worse health than those who did not respond to the travel questions but responded to the HAQ, EQ-5D VAS, and EQ-5D TTO (all *P* < 0.01). No difference could be observed between respondents and noncompleters in terms of gender (*P* = 0.29), duration of RA (*P* = 0.44), or BMI (*P* = 0.26).

During the three-month period, the 2847 patients reported on average 2.8 (sd 4.0; median 2) visits to a rheumatology outpatient clinic for consultations with doctors or nurses, blood tests, and collection of medication (Table 2). The median visits frequency was two while 25% of the sample had three or more visits during the three-month period. Average travel cost per visit to the rheumatology clinic was = 13 € (sd 20 €). 1144 patients (40%) had at least one visit to their general practitioner and this group had on average 2.3 visits with the general practitioner during the three-month period. As expected, mean travel costs were lower for visits to general practitioners (4 €) and private medical specialists (10 €) than to outpatient clinics (11–13 €) and hospital admissions (16 €).

Many (63%) patients travelled to the health provider by private car, while 11% used bus, train, or metro, and 7% walked or biked (Table 3). Fewer than 5% indicated that they used transport services provided by the hospital. The average travel time to the rheumatology outpatient clinic was 73 minutes per visit.

Table 4 provides estimates of the aggregated average three-month travel costs and time for all health care providers. The average patient had 4.4 (sd 5.7) contacts with health care providers during the three-month period and spent 4.6 hours and 56 € when travelling to and from the health care providers. The mean contact frequency, mean travel time, and mean costs were similar for various subgroups, although the average number of contacts was higher for women, those living in larger cities, those out of the labor market, and those on biological treatment. Average travel times were higher for patients aged 50–75 years, out of the labor market, with elevated CRP level, and on biological treatment. Mean travel costs were higher for patients living in larger cities, with elevated CRP level, and on biological treatment. Patients from a single hospital (with a large rural catchment area) reported statistically significant higher travel time and expenses (data not shown).

In regression analyses, none of the patient characteristics correlated strongly with the number of contacts, travel time or costs, and only a small proportion of the variation could be explained by such models (*R* ^2^ < 0.03) (data not shown). A larger proportion of the variation was explained by the regression models when the number of contacts with health care providers was included as an explanatory variable, although none of the other estimated parameters reached statistical significance. These results were unchanged when variables for hospitals were introduced and when the analysis was conducted separately for each hospital (data not shown).

## 4. Discussion

In this study, we obtained data on patients' travel time and costs using a self-complete questionnaire developed for the purpose. Patients with RA attending at least one outpatient clinic for treatment within a 3-month period had on average 4.4 contacts with health care providers and spent 4.6 hours and 56 € on transport to and from these providers. The majority of the contacts were to the rheumatology outpatient clinic (on average 2.8 times during the three-month period, 3.4 hours, and 41 €). The most frequent mode of travel was private car, thereafter public bus, train, or metro. During the same period of time, nearly 40% of the patients also visited their general practitioners and those that did had on average 3.2 contacts during three-month period and spent about one hour and 6 € DKK on travel to their GP.

Overall, the costs and amount of time that these patients spent on travel in connection with health care treatment are not surprising. Frequent contacts with health care providers use more of patients' time and increase travel expenses. We were unable to identify strong associations between travel time and costs and patient characteristics, and we found significant variations at only one of the participating hospitals.

Social and demographic factors had relatively little influence on travel time and costs for rheumatic patients. RA patients living in larger cities, with elevated CRP level, and on biological treatment had more contacts and spent more time and expense on travels than other patients, but the association was not particularly strong and these variables were unable to explain much of the observed variation in travel time and costs. One hypothesis could be that patients living in larger cities are more likely to respond to symptoms than patients from rural areas.

We expected greater variation in travel time and expense between different population groups and disease characteristics. It may be that none of the variables tested have any influence on patients' travel time and costs. Thus, although travel time and costs are influenced by the distance between the patient's residence and the health provider, the geographical distribution of providers may be broadly similar (at least in Denmark), reflecting fairly equal access to care. Another explanation might be that variations in travel time and costs are large, and this study sample had insufficient statistical power to identify relevant associations. The sample size was considerable (*n* = 2847); however, we do not consider this a primary explanation.

We collected self-reported travel costs for contacts to different health care providers. There were no problems reported in understanding what information was requested, but a considerable proportion (8%) did not answer the transport questions. Lack of knowledge about the exact travel time and costs is the most likely explanation, but the questions may have been unclear for some respondents—particularly for those using private car as the mode of travel. Here they would have needed to take into account the distance driven, fuel costs, and other running costs of the car, and not all patients may have known these. Public transport such as bus, train, and metro is easier to cost as a certain amount is paid for the ticket. Judging from the distribution of the cost data, however, there appeared to be a reasonable relation between travel time and travel cost.

An alternative to the focus on travel costs could have been to ask patients to provide information only on travel distance, as done in other studies [10–13]. Patients are likely to have a fairly good idea of the distance to the health care provider, but this approach would give less precise estimates of costs based on distance traveled. While transport by private car and taxis could be estimated by applying a predetermined cost per km, the cost related to bus, trains, and metro would be more complicated and would need a detailed knowledge of the fee structure of these transport modes.

The frequency of visits to the six types of health care providers appeared to be straightforward to answer. The 3-month interval was chosen to be able to make a reasonable estimate of the annual cost and was short enough that patients would remember a visit and the transport mode. We interpreted missing replies to indicate no use of health providers within the previous three months. In future studies, it might be considered to ask patients to indicate zero as an indication of no contacts.

We have no exact way of validating the travel time and cost estimates. However, the detailed data on travel time fits well with expectations based on the relatively dense distribution of health care facilities within a relatively small country like Denmark. Also, our sample came from 11 of the 28 outpatient rheumatology hospital departments in Denmark. We observed significant variations in travel time for only one hospital.

Unlike several other studies on patients travel costs [10–12], we decided not to assign a monetary value on travel time, as the data collection did not include a measure of value of time or income of the patients. We did have information on education and labor market attachment and could have imposed a stratified measure of hourly income obtained from the national statistics bureau. However, we should ideally use a measure of opportunity cost and that is less simple to obtain, as it would require information about the patient's time use if they had not been visiting a health care provider. If we followed the recommendations of the human capital approach and assumed that all contacts with health care providers should be valued according to the opportunity cost of leisure time (personal income net of tax), we would have derived at an average cost of transport corresponding to 95 € per patient per three-month period.

In this analysis, we have focused solely on the travel costs of the patients. Some studies have also considered the travel costs of accompanying persons. From an economic perspective, the relevance of including the cost of accompanying persons can be discussed and it will be highly dependent on the individual patient's need to be accompanied. In the case of most RA patients in outpatient care, we assume that an accompanying person is not required.

In this study, we relied on patients self-report of the travel time and cost. More than 70% of the eligible patients responded to the travel time and cost questions. However, a substantial proportion of the patients (8.3%) did not provide valid response. There are potential biases in self-report data. Recall bias may be important as we asked patients to report their use of health care services during a three-month period. There may be some element of under- or overreporting although it is difficult to assess the direction and consequences. The explanation to missing response is difficult to assess. We applied a fairly simplistic approach to missing items although more advanced methods could have been employed (e.g., multiple imputation). Such a more advanced approach would ensure larger variability in the data in comparison with the approach we used. However, a crucial prerequisite for assessment is some explanation as to the pattern of missing data. Here we assumed that those patients who did not provide valid responses may be represented by those who provided valid response. A brief inspection using logistic regression to identify different social characteristics in those with and without valid responses did suggest a systematic pattern in patients with missing responses.

Another potential bias is the fact that out of the 3700 patients identified eligible for the study, 15 percent did not respond to the questionnaire at all and further 8 percent who responded to the questionnaire did not provide answers on the travel time and cost questions. We were able to identify difference between responders and nonresponders, and it appeared that responders were younger, more likely to be married/cohabiting, living in a city area, had longer education, and were in the labor market. Respondents were also in less severe health states as nonresponders. It is difficult to assess the consequences of such systematic pattern in the nonresponders, but it may provide some explanation as to the reasons for non-response, namely, that information about travel time and cost may not be relevant or important and therefore patients have avoided providing answers on these questions.

## 5. Conclusions

The results of this study suggest that patients with rheumatoid arthritis in active outpatient care have on average 4.4 contacts with health care providers within a 3-month period and spend 4.6 hours and 56 € on travelling to and from these providers. Patients with RA thus have private travel time and costs associated with treatment. These findings are important when analyzing the social impact of treatment for patients with rheumatoid arthritis.

## Figures and Tables

**Table 1:** Characteristics of patients with rheumatoid arthritis (RA) invited to complete a survey including questions on travel costs to health providers.

	Individuals invited to survey (*n* = 3704)					
	Completed travel questions (*n* = 2847)		Returned questionnaire but did not complete travel questions (*n* = 308)		Did not return the questionnaire (*n* = 549)	
Gender						
Male	708	25%	85	28%	131	24%
Female	2139	75%	223	72%	418	76%
Age group (yrs.)						
≤50	679	24%	18	6%	126	23%
51–75	1868	66%	209	68%	269	49%
>76	300	11%	81	26%	154	28%
Cohabiting						
Single	932	33%	144	47%	NA	
With partner	1915	67%	164	53%	NA	
Residential area						
Rural/small towns	1832	64%	219	71%	340	62%
Larger cities	1015	36%	89	29%	209	38%
Education						
Short or none	710	33%	117	61%	NA	
Medium term	1047	49%	131	69%	NA	
Long	1090	51%	60	31%	NA	
In the labor market						
Yes	981	34%	30	10%	NA	
No	1672	59%	220	71%	NA	
Missing data	194	7%	58	19%	NA	
Disease duration (RA)						
0–2 years	684	24%	64	21%	124	23%
3–10 years	1136	40%	127	41%	196	36%
10+ years	1027	36%	117	38%	229	42%
C-reactive protein level						
Normal	1998	70%	199	65%	350	64%
Elevated	849	30%	109	35%	199	36%
Body mass index (BMI)						
Underweight	106	4%	8	3%	NA	
Normal	1531	54%	178	58%	NA	
Overweight	832	29%	91	30%	NA	
Obese	378	13%	31	10%	NA	
Biological medicine						
No	2249	79%	264	86%	464	85%
Yes	598	21%	44	14%	85	15%
Health score (mean, sd)						
Global VAS (*n* = 2847; 308; 549)	35	(25)	34	(24)	36	(7)
HAQ score (*n* = 2846; 308)	0.7	(0.6)	0.6	(0.6)	NA	
EQ-5D VAS (*n* = 2520; 158)	40	(28)	54	(33)	NA	
EQ-5D index (*n* = 2520; 158)	0.307	(0.421)	0.453	(0.468)	NA	

Notes: VAS: visual analog scale; HAQ: health assessment questionnaire; NA: not available.

**Table 2:** Frequency of visits to health care providers in a 3-month period and average travel costs per visit (return trip) as reported by patients with rheumatoid arthritis.

	*n*	Mean	Sd.	p 25	p 50	p 75
Rheumatoid outpatient clinic						
Number of visits	2847	2.8	4.0	1.0	2.0	3.0
Travel expenses (return) (€)	1692	13.3	19.5	3.1	7.7	15.4
Other outpatient clinics						
Number of visits	482	2.1	2.8	1.0	1.0	2.0
Travel expenses (return) (€)	328	10.9	19.8	0.0	5.6	13.1
General practitioner						
Number of visits	1144	2.3	2.7	1.0	2.0	3.0
Travel expenses (return) (€)	742	4.2	10.5	0.0	1.5	4.6
Private medical specialist						
Number of visits	407	1.4	1.3	1.0	1.0	2.0
Travel expenses (return) (€)	247	9.7	13.7	0.0	5.1	11.7
Hospital admission						
Number of visits	249	1.3	4.3	0.0	1.0	1.0
Travel expenses (return) (€)	124	16.1	33.0	0.0	3.1	16.0
Emergency department						
Number of visits	156	0.6	1.0	0.0	0.0	1.0
Travel expenses (return) (€)	68	3.7	6.4	0.0	0.0	4.6

Note: Sd: standard deviation; DKK: Danish crowns; p  25, p 50, p 75 indicate percentiles. Travel expenses reported in 2013-€.

**Table 3:** Mode of travel and mean (return) travel time to different health care providers as reported by patients with rheumatoid arthritis.

Mode of travel	Walking, biking	Bus, train, metro	Hospital transport	Taxi	Private car	Ambulance	All
Rheumatology outpatient clinic							
Number of patients	198	324	131	68	1796	18	2416
% of sample	7.0%	11.4%	4.6%	2.4%	63.1%	0.6%	84.9%
Mean travel time (min.)	33	81	101	69	69	110	73
Other outpatient clinics							
Number of patients	40	53	32	12	366	4	489
% of sample	1.4%	1.9%	1.1%	0.4%	12.9%	0.1%	17.2%
Mean travel time (min.)	34	70	120	43	59	78	64
General practitioner							
Number of patients	308	89	15	34	824	3	1247
% of sample	10.8%	3.1%	0.5%	1.2%	28.9%	0.1%	43.8%
Mean travel time (min.)	20	40	41	26	24	33	25
Private medical specialist							
Number of patients	32	35	11	12	233	3	320
% of sample	1.1%	1.2%	0.4%	0.4%	8.2%	0.1%	11.2%
Mean travel time (min.)	25	64	101	51	59	123	59
Hospital admission							
Number of patients	4	9	23	16	127	25	192
% of sample	0.1%	0.3%	0.8%	0.6%	4.5%	0.9%	6.7%
Mean travel time (min.)	16	102	89	50	67	33	69
Emergency department							
Number of patients	8	6	2	7	86	2	109
% of sample	0.3%	0.2%	0.1%	0.2%	3.0%	0.1%	3.8%
Mean travel time (min.)	16	55	31	29	43	25	41

**Table 4:** Three-month average travel time and costs for patients with different characteristics.

	*n*	Number of contacts		Travel time (min.)		Travel cost (€)	
		Mean	(Sd)	Mean	(Sd)	Mean	(Sd)
All	2847	4.4	(5.7)	277	(516)	56	(148)
Gender							
Male	708	**3.9**	**(3.9)**	254	(469)	50	(89)
Female	2139	**4.6**	**(6.2)**	284	(531)	58	(163)
Age group (yrs.)							
≤50	679	4.3	(3.7)	**236**	**(304)**	56	(111)
51–75	1868	4.5	(6.4)	**292**	**(574)**	56	(152)
>76	300	4.1	(4.6)	**271**	**(514)**	56	(189)
Cohabiting							
Single	932	4.3	(7.1)	279	(532)	57	(185)
With partner	1915	4.5	(4.9)	276	(509)	56	(126)
Residential area							
Rural/small towns	1832	**4.2**	**(6)**	264	(494)	**52**	**(127)**
Larger cities	1015	**4.7**	**(5.1)**	299	(554)	**64**	**(180)**
Education							
Short or none	710	4.1	(4.4)	269	(521)	54	(146)
Medium term	1047	4.4	(4.2)	276	(490)	54	(83)
Long	1090	4.7	(7.4)	282	(538)	60	(191)
In the labor market							
Yes	981	**4.1**	**(6.6)**	**219**	**(368)**	52	(158)
No	1672	**4.6**	**(4.7)**	**300**	**(528)**	58	(142)
Missing data	194	4.9	(8.2)	364	(893)	62	(144)
Disease duration (RA)							
0–2 years	684	4.6	(4.2)	269	(464)	56	(101)
3–10 years	1136	4.1	(4.9)	272	(565)	49	(133)
10+ years	1027	4.7	(7.2)	288	(493)	64	(186)
CRP level							
Normal	1998	4.3	(4.7)	**263**	**(462)**	**52**	**(117)**
Elevated	849	4.7	(7.6)	**309**	**(624)**	**65**	**(203)**
BMI							
Underweight	106	5.0	(4.6)	319	(388)	52	(58)
Normal	1531	4.3	(6.4)	265	(490)	54	(152)
Overweight	832	4.3	(4.4)	275	(496)	58	(147)
Obese	378	4.9	(5.2)	316	(671)	64	(150)
Biological medication							
No	2249	**4.3**	**(5.9)**	**260**	**(499)**	**53**	**(145)**
Yes	598	**4.9**	**(4.9)**	**339**	**(573)**	**69**	**(157)**

Note: bold figures indicate *P* < 0.05 (ANOVA); CRP: C-reactive protein; BMI: body mass index. Travel expenses reported in 2013-€.
